# Examining patient trust towards physicians between clinical departments in a Chinese hospital

**DOI:** 10.1371/journal.pone.0259945

**Published:** 2021-11-29

**Authors:** Judy Yang, Yuanzheng Lu, Xiaoxing Liao, Mary P. Chang

**Affiliations:** 1 Department of Anesthesiology, Columbia University, New York, NY, United States of America; 2 Seventh Affiliated Hospital, Sun Yat-sen University, Shenzhen, China; 3 Department of Emergency Medicine, UT Southwestern Medical Center, Dallas, TX, United States of America; Imam Abdulrahman Bin Faisal University, SAUDI ARABIA

## Abstract

The purpose of this cross-sectional survey study is to quantitatively examine the differences in patient trust towards physicians between four different clinical departments in a Chinese hospital. Using a validated modified Chinese version of the Wake Forest Physician Trust Scale, we measured patient trust in each department, and also collected data on patient demographics. A total of 436 patients or family members were surveyed in the departments of emergency medicine, pediatrics, cardiology, and orthopedic surgery. Significant differences were found between the departments, especially between pediatrics (trust score 43.23, range 11–50) and emergency medicine and cardiology (trust scores 45.29 and 45.79, respectively with range of 11–50). The average total score across all four departments was 44.72. There are indications that specifically comparing departments, such as patient demographics or department structure, could be helpful in tailoring patient care to improve physician-patient relationships.

## Introduction

Trust has always been valued in any relationship, especially between the patient and physician. However, cases of violence plaguing Chinese hospitals have been making headlines since the early 2000’s. Over the first decade of the 21^st^ century to the end of 2011, 124 incidents of violence has been reported on media, including 29 murders and 52 serious injuries [1]. Cases of stabbings, acid being poured on physician faces, and hospital explosions raid the news pages, making these cases all too common. In 2014, the incidences of extreme violence against medical personal tallied over 150 [2]. These incidents no doubt play a role in the weariness of the young workforce to enter the medical profession. According to the Chinese Medical Doctors Association, 11% of physicians hoped their children would follow their footsteps in 2001 and only 7% in 2011 [3]. With the rising need of China’s patients, the dearth of physicians couldn’t come at a worse time: as cited in China’s Health Statistics Yearbook 2012, the increase in the number of patients visiting Chinese hospitals from 4 billion in 2005 to 6.2 billion in 2011 was only met with an increase in physicians of only 170,000 between 2008 and 2011 [4].

With the public and media bringing more attention to the subject of mistrust between patients and physicians, researchers have begun to look more closely at the critical issue hindering public health. Driving forces of patient-physician mistrust and solutions have been suggested through both quantitative and qualitative studies that assess patient trust in physicians across hospitals in China [5–7]. Studies have looked at correlations of trust with patient demographics such as age, education level, annual income, and health insurance coverage across hospitals, with other studies aiming to identify determinants of patient trust in physicians [5,8].

No studies have yet looked at data specifically between hospital departments. Future physicians should not have to consider their safety when deciding their future specialty, just as no patients seeing specific specialties should ever have to suspect their quality of care. Thus, such a study has the potential to help appropriately tailor solutions to individual departments and allow hospitals to provide a safe environment for both physician and patient to work together and achieve greater health.

The Chinese version of the Wake Forest Physician Trust Scale (C-WFPTS) has been determined to have good psychometric properties, reliability, and validity [9]. In this cross-sectional study, we aimed to quantitatively examine the differences in patient trust towards physicians between several departments within a hospital in China using a modified C-WFPTS.

## Methods

### Ethics

The survey study was conducted at the 7^th^ Affiliated Hospital, Sun Yat-Sen University in Shenzhen, China between April to June 2019. The 7^th^ Affiliated Hospital is classified as a suburban, tertiary hospital with an annual outpatient volume of approximately 220,000 patients and inpatient volume of approximately 12, 000 patients as of 2018. This research was approved by the Research and Experimental Animal Ethics Committee, The Seventh Affiliated Hospital of Sun Yat-sen University and the University of Texas at Southwestern Institutional Review Board.

### Survey

The survey consists of questions including demographics, education level, socioeconomic status, insurance type, questions regarding patient perception of care, and the C-WFPTS (S1 Appendix). The survey was translated to Mandarin by two native Mandarin speakers and verified by a third native speaker. No patient identifiers were recorded and to ensure there was no duplication of patients in our dataset, the research team member asked the patient if they have taken the survey before. The possible scores on the C-WFPTS are between 11 and 50.

### Survey implementation

A research team member who was fluent in Mandarin surveyed patients in outpatient clinics of emergency medicine, pediatrics, cardiology, orthopedic surgery between April to June 2019. The surveys were administered after patients were seen by their physicians or if they have visited the specific department before. If the patient was less than 18 years of age, the parent was surveyed. Moreover, for patients over the age of 18 who requested that their family members take the survey for them, we gave the survey to the family member if they were familiar with the patient’s treatment process. Patients gave oral consent to be enrolled in the study. Inclusion criteria were any patients who understood and spoke Mandarin and provided consent. There was no exclusion criterion.

### Data analysis

We used Microsoft Excel for data collection and SAS (Cary, NC) for statistical analysis. The significance level used is p < 0.05. Overall descriptive statistics for demographics was calculated as well as between departments. Data were analyzed for correlations between patient demographic and trust and the ANOVA test was used for inter- department comparison. Patient trust answers were converted to a Likert scale of 1 to 5 and calculated as ordinal data.

## Results

### Demographic characteristics of sampled patients

The survey was completed by a total of 436 patients. We surveyed 113 patients in emergency medicine, 112 patients in pediatrics, 107 patients in cardiology, and 104 patients in orthopedic surgery. 47.02% of the total surveyed patients were female and the average age was 38.81 years Table 1.

**Table 1:** Demographic characteristics of sampled patients in each department.

Variables	Overall (%)	Emergency Medicine (%)	Pediatrics (%)	Cardiology (%)	Orthopedic Surgery (%)
**age (years)**					
18–29	89 (20.5)	40 (35.4)	24 (21.8)	5 (4.7)	20 (19.2)
30–39	168 (38.7)	37 (32.7)	68 (61.8)	26 (24.3)	37 (35.6)
40–49	97 (22.4)	24 (21.2)	17 (15.5)	33 (30.8)	23 (22.1)
50–59	53 (12.2)	9 (8.0)	-	23 (21.5)	21 (20.2)
60–69	25 (5.8)	3 (2.7)	1 (0.9)	19 (17.8)	2 (1.9)
>/ = 70	2 (0.5)	-	-	1 (0.9)	1 (1.0)
**Gender**					
Male	231 (53.0)	65 (57.5)	42 (37.5)	65 (60.7)	59 (56.7)
Female	205 (47.0)	48 (42.5)	70 (62.5)	42 (39.3)	45 (43.3)
**Forms filled by family member**	183 (42.0)	28 (24.8)	112 (100.0)	23 (21.5)	20 (19.2)
**Age of family member (patient)**					
<1 month	1 (0.6)	-	1 (0.9)	-	-
1 month-<2 years	36 (19.7)	-	36 (32.1)	-	-
2–11 years	76 (41.5)	2 (7.1)	72 (64.3)	-	2 (10.0)
12–15 years	7 (3.8)	4 (14.3)	3 (2.7)	-	-
16–17 years	4 (2.2)	3 (10.7)	-	-	1 (5.0)
18–29	-	-	-	-	-
30–39	3 (1.6)	2 (7.1)	-	-	1 (5.0)
40–49	2 (1.1)	2 (7.1)	-	-	-
50–59	19 (10.4)	6 (21.4)	-	7 (30.4)	6 (30.0)
60–69	22 (12.0)	9 (32.1)	-	7 (30.4)	6 (30.0)
>/ = 70	13 (7.1)	-	-	9 (39.1)	4 (20.0)
**Education level**					
Primary school or less	78 (17.9)	15 (13.3)	11 (9.8)	36 (33.6)	16 (15.4)
High school completed	121 (27.8)	32 (28.3)	26 (23.2)	24 (22.4)	39 (37.5)
Bachelor degree completed	201 (46.1)	59 (52.2)	62 (55.4)	41 (38.3)	39 (37.5)
Professional degree completed	36 (8.3)	7 (6.2)	13 (11.6)	6 (5.6)	10 (9.6)
**Marital status**					
Never	58 (13.3)	33 (29.2)	-	6 (5.6)	19 (18.3)
Married	370 (84.9)	78 (69.0)	112 (100)	97 (90.7)	83 (79.8)
Divorced	8 (1.8)	2 (1.8)	-	4 (3.7)	2 (1.9)
Widowed	-	-	-	-	-
**Job status**					
Employed	324 (74.5)	90 (79.6)	83 (74.8)	70 (65.4)	81 (77.9)
Unemployed	71 (16.3)	16 (14.2)	26 (23.4)	16 (15.0)	13 (12.5)
Retired	40 (9.2)	7 (6.2)	2 (1.8)	21 (19.6)	10 (9.6)
**Occupation type**					
Professional/managerial	243 (56.0)	70 (61.9)	69 (62.2)	53 (50.0)	51 (49.0)
Other non-manual	65 (15.0)	19 (16.8)	14 (12.6)	12 (11.3)	20 (19.2)
Agricultural	13 (3.0)	3 (2.7)	1 (0.9)	6 (5.7)	3 (2.9)
Manual labor	35 (8.1)	6 (5.3)	6 (5.4)	10 (9.4)	13 (12.5)
None	78 (18.0)	15 (13.3)	21 (18.9)	25 (23.6)	17 (16.4)
**Home ownership**					
Own	258 (59.2)	62 (54.9)	62 (55.4)	71 (66.4)	63 (60.6)
Rent	178 (40.8)	51 (45.1)	50 (44.6)	36 (33.6)	41 (39.4)
**Household registration**					
Urban	274 (62.8)	75 (66.4)	70 (62.5)	68 (63.6)	61 (58.7)
Rural	162 (37.2)	38 (33.6)	42 (37.5)	39 (36.4)	43 (41.3)
**Insurance**					
Private	23 (5.3)	5 (4.4)	9 (8.0)	4 (3.7)	5 (4.8)
Shenzhen	257 (58.9)	53 (46.9)	74 (66.1)	66 (61.7)	64 (61.5)
Non-Shenzhen	50 (11.5)	18 (15.9)	2 (1.8)	15 (14.0)	15 (14.4)
Uninsured	30 (6.9)	11 (9.7)	4 (3.6)	8 (7.5)	7 (6.7)
Multiple	76 (17.4)	26 (23.0)	23 (20.5)	14 (13.1)	13 (12.5)
**Monthly income (RMB)**					
<5,000	163 (37.8)	38 (33.9)	30 (27.0)	53 (50.5)	42 (40.8)
5000–10,000	147 (34.1)	44 (39.3)	46 (41.4)	23 (21.9)	34 (33.0)
10,000–20,000	80 (18.6)	20 (17.9)	24 (21.6)	20 (19.0)	16 (15.5)
>20,000	41 (9.5)	10 (8.9)	11 (9.9)	9 (8.6)	11 (10.7)
**Medical diagnosis**					
Present	302 (69.3)	95 (84.1)	31 (27.7)	92 (86.0)	84 (80.8)
None	134 (30.7)	18 (15.9)	81 (72.3)	15 (14.0)	20 (19.2)
**Known physician status**					
Known	244 (56.0)	20 (17.7)	80 (71.4)	70 (65.4)	74 (71.2)
Unknown	192 (44.0)	93 (82.3)	32 (28.6)	37 (34.6)	30 (28.8)
**Known physician status**					
Resident	60 (24.6)	6 (30.0)	25 (31.3)	10 (14.3)	19 (25.7)
Attending	98 (40.2)	11 (55.0)	37 (46.2)	32 (45.7)	18 (24.3)
Associate chief	42 (17.2)	1 (5.0)	18 (22.5)	1 (1.4)	22 (29.7)
Chief	83 (34.0)	2 (10.0)	32 (40.0)	29 (41.4)	20 (27.0)
**Number of visits**					
Once	158 (37.9)	47 (50.0)	19 (17.0)	36 (33.6)	56 (53.8)
Multiple	259 (62.1)	47 (50.0)	93 (83.0)	71 (66.4)	48 (46.2)

### C-WFPTS outcomes

Assessment of patient trust using the original 11 items of the C-WFPTS showed that the average patient to physician trust among all four departments were 45.29 in emergency medicine, 43.23 in pediatrics, 45.79 in cardiology, and 44.61 in orthopedic surgery, with an overall average of 44.72 (Table 2). The overall mean Likert score was 4.07, standard deviation 0.50. The mean Likert scores of the individual departments are: 4.11 (SD 0.50) in emergency medicine, 3.93 (SD 0.44) in pediatrics, 4.16 (SD 0.45) in cardiology, and 4.06 (SD 0.57) in orthopedic surgery. ANOVA test showed that trust among patients in pediatrics was significantly lower than those in cardiology (p<0.01) and emergency medicine (p<0.05), without any significant difference between the other departments.

**Table 2:** Trust score by department.

Trust Score	21-Nov	22–32	33–43	44–54	55	Total patients	Average score
**Emergency Medicine**	-	2 (1.8%)	42 (37.2%)	65 (57.5%)	4 (3.5%)	113	45.29
**Pediatrics**	-	1 (0.9%)	56 (50.0%)	53 (47.3%)	2 (1.8%)	112	43.23
**Cardiology**	-	-	32 (29.9%)	69 (64.5%)	6 (5.6%)	107	45.79
**Orthopedic surgery**	1 (0.96%)	2 (1.9%)	38 (37.6%)	56 (53.9%)	7 (6.7%)	104	44.61
**Total**	1	5	167	238	19	436	

### Trust score and reasons for mistrust

Among the 436 patients surveyed, 7 patients were willing to provide reasons for mistrust towards physicians, with 5 given by patients surveyed in the pediatrics department and the remaining 2 from emergency medicine and cardiology. Of the 7 reasons given, 2 were due to lack of improvement in condition, 4 were due patient dissatisfaction towards the hospital, and 1 was due to dissatisfaction with a physician seen at a different department. In addition, 2 reasons were given by patients whose trust scores ranged from 22–32, 3 from patients with trust scores from patients with trust scores 33–43, and 2 from patients with trust scores that ranged from 44–54.

## Discussion

The frequency of medical disputes in China and need to repair the lack of trust between physician and patient has been the catalyst for many recent studies set on finding causes of mistrust and government reforms on medical education and training, health insurance coverage, and primary care facilities. Specifically, among the many incidences of conflict, the frequency of medical disputes is higher in the pediatrics department than that in other specialties and no doubt plays a role in the shortage of pediatricians that has sent the country’s leaders looking for remedies to avoid an endless cycle of discontent [10,11]. In addition, with the relatively recent introduction and development of emergency departments to Chinese public hospitals, emergency departments are also faced with combatting overcrowding and rising patient dissatisfaction [12]. Thus, a search for targeted solutions for individual departments may be beneficial to the more universal reforms already taking place.

In this study, we used a modified Chinese version of the Wake Forest Physician Trust Scale, measuring patient trust in each department and collecting data on patient demographics in four separate departments, in order to examine the differences in patient trust towards physicians between. The results suggest that patient trust is significantly lower in the pediatrics department than in both emergency medicine and cardiology. This comes as no surprise due to the higher frequency of medical disputes in the pediatrics department. As suggested by Xu & Zhang (2014), families are more sensitive to the health of their child due to the one-child policy, and physicians are often the first to be blamed for any lack of improvements in their child’s condition [10]. Nevertheless, the few reasons for mistrust given by patients in the pediatrics department may help point to feasible changes that can be taken by public hospitals. Most of the reasons given by parents concerned the hospital rather than the physician itself. One theme of patient concerns in the structure of their hospital care is the hospital environment and facilities [13]. In this study, respondents made comments specifically to the lack of masks accessible for their children and separation from other potentially infection-transmitting patients, lack of a pediatrics emergency department, and inconvenient weekend times. The additional reasons given unrelated to hospital structure concerned the lack of improvement of their child’s condition despite multiple visits. It would be interesting to conduct a longitudinal study that follows the changes of the emergency department and hospital over time in terms of patient demographics and trust.

However, what was interesting was the relatively higher patient trust in emergency medicine. In comparison to a more-established tertiary hospital like Peking Union Medical College Hospital, with a reported average annual outpatient volume of 3.264 million and whose emergency department was established in 1983, the Seventh Affiliated Hospital of Sun Yat-sen University is relatively new, having just opened for one year, with an annual outpatient volume of 220,000 [14]. Thus, the emergency department in which surveys were distributed for this study is still in the initial stages of development and not as riled with the dilemmas of overcrowding, delayed admissions and attendance as a more developed emergency department would. Studies have indicated that emergency departments faced longer patient waiting times and more medical disputes and complaints with increased development [12]. Additionally, according to a study on waiting times and patient satisfaction in an endocrinology outpatient clinic, patients that experience longer waiting times consider their medical service as less accessible and correlate with less satisfaction, regardless of the time actually spent with the physician [15]. Since the emergency department scores were higher than other departments, future research can evaluate for factors that contribute to a higher score.

Overall, the average total score was 44.72 and mean Likert score was 4.07. Previous studies that likewise used the C-WFPTS found a mean Likert score of 3.35 among hospitals in Shandong, Jiangsu, Hubei, Henan, and Sichuan provinces and an average total score of 53.83 among hospitals in Shanghai [8,9]. One obvious difference between these studies that could account for such differences is the province that these hospitals are located in. Hospital reputation, morals of medical staff, and caring attitudes and emotional support are major themes identified by patients that affect their perceptions of their hospital care [13]. Thus, differences in culture and social perceptions among both patients and physicians in individual provinces could affect these important factors. It would be interesting to further compare the results of this study to other tertiary hospitals in the Guangdong province using the C-WFPTS.

Additionally, there are demographic differences between the four departments. In a similar study consisting of 12 Chinese hospitals looking at factors influencing trust towards physicians, it was found that respondents who were young and had higher levels of education and income tended to be less satisfied with their physicians [8]. It can be noted of the respondents surveyed in our study, a higher percentage of respondents under the age of 40 years (84%), who received a bachelor degree (55%), and who earned over 5,000 yuan monthly (73%) were surveyed in the pediatrics department. Thus, there are indications to further identify significant subpopulations specific to each department in order to tailor medical care services to better patient trust and satisfaction.

As other studies that have used the Wake Forest Physician Trust Scale or a modified version of it, this study is subject to the limitations of social tendencies to provide culturally appropriate responses [9]. Despite the emphasis that information will not be shared and that all patient responses will remain anonymous, there was often noticeable hesitation from patients when given the survey. Thus, it is also possible that answering “uncertain” to survey items could be an alternative to avoid giving a negative response. Furthermore, patients unsatisfied with treatment could have refused to participate due to their dissatisfaction. Finally, improvements that could be made to this study would be to provide a larger sample size and to survey multiple hospitals longitudinally.

## Conclusion

From the beginnings of healthcare, physicians strive to establish trust with their patients. In this study of a Chinese hospital, differences in the level of patient trust exist between various departments, whose respondents also differ in their patient demographics. Further research may also be warranted to understand where the mistrust stems from, the institutions, or the physicians themselves, so that the future of healthcare in China can minimize issues of trust, deliver better patient-centered care and encourage more of their citizens to join the diminishing health workforce.

## Supporting information

S1 Appendix Patient demographics and trust survey in English and Chinese. (DOCX)
