# Supplementary material for: Examining patient trust towards physicians between clinical departments in a Chinese hospital
Source: PLoS One. 2021 Nov 29;16(11):e0259945. doi: 10.1371/journal.pone.0259945 (PMC8629292; doi:10.1371/journal.pone.0259945)
Supplement: S1 Appendix — (DOCX) [file pone.0259945.s001.docx]

**Appendix 1**. Patient demographics and trust survey in English and Chinese.

1. What is your age (years)? ______ 年纪：______
2. What is your sex? Male Female 性别：______
3. If you are a parent filling out this survey for your child, how old is your child? __________ 如果你是父母替儿女填表，你的孩子年纪是 _______
4. What is the highest level of education you have obtained? 您最高的学历？
   1. No education or finished primary school 无学历或完成小学学业
   2. Finished high school 完成高中学业
   3. Finished college 完成大学学业
   4. Professional degree 职业学校学位
5. Marital status 婚姻状况
   1. Never married 未婚
   2. Married 已婚
   3. Divorced 离婚
   4. Widowed 寡居
6. Job status 职业现状
   1. Employed 工作中
   2. Unemployed 无工作
   3. Retired 退休/下岗
7. Occupation type职业种类
   1. Professional/managerial 专业/管理
   2. Other non-manual 其它非劳力工作
   3. Agricultural 农业
   4. Manual laborer 劳工
   5. None 无
8. Home ownership 房屋居住状况
   1. Own 拥有房产
   2. Rent 租住
9. Rural-urban status/registration/hukou户口登记
   1. Urban 城市
   2. Rural 乡村

10. Insurance 保险

- - 1. Private insurance 商业保险
    2. Shenzhen social insurance 深圳医保
  1. Non-Shenzhen social insurance 外地医保
  2. Uninsured 无保险

1. Approximate monthly income 每月收入

- < 5,000 RMB 人民币
- 5,000 - 10,000 RMB 人民币
- 10,000 - 20,000 RMB 人民币
- > 20,000 RMB 人民币

1. Do you have any medical diagnoses (If you are the parent, please fill out your child’s)

你有医疗诊断吗？（如果你是父母，请填写你孩子的医疗诊断）

____________________________________________________________________________________________________________________________________________________________

1. Do you know your physician’s status? Yes/No

你是否知道就诊医生的职称？是/否

- Resident physician 住院医师
- Attending physician 主治医师
- Associate chief physician/associate professor 副主任医师/副教授
- Chief physician/professor 主任医师/教授

1. How many times have you visited this department? 第几次来这科室？

- First time 第一次
- Multiple times 多次

| Your Possible Attitudes  你认同下列描述的程度 | Strongly Agree 非常认同 | Agree 认同 | Disagree 不认同 | Strongly Disagree  非常不认同 | Uncertain 不确定 |
| --- | --- | --- | --- | --- | --- |
| For the sake of my health, my doctor will do whatever I need.  为了我的健康，我的医生会为我尽力而为 |  |  |  |  |  |
| My doctor always cares more about what is convenient for him/her than about my medical needs.  我的医生总是为着他自己的方便，而不是为我着想 |  |  |  |  |  |
| My doctor’s competence level does not achieve the degree I expected.  我的医生的能力没有达到我的期望 |  |  |  |  |  |
| My doctor is extremely thorough and careful.  我的医生非常仔细和完善 |  |  |  |  |  |
| I think my doctor’s treatment decisions are best for me.  我相信我的医生的治疗是对我最佳的 |  |  |  |  |  |
| My doctor will explain honestly to me about the different treatment options available for me currently.  我的医生会诚实的解释现有的不同疗法 |  |  |  |  |  |
| Sometimes my doctor does not pay full attention to what I am trying to tell him/her.  有时候我的医生没有完全注意听我对他说的病情 |  |  |  |  |  |
| I feel my doctor will release my personal information to unauthorized persons  我觉得我的医生会把我的个人资料泄露给别人 |  |  |  |  |  |
| My doctor will act in my interests, not in his/her or the hospital’s interests.  我的医生会为我的利益着想，而不是为他或医院的利益着想 |  |  |  |  |  |
| I don’t hesitate to put my life to my doctor’s hands.  我不会犹豫将我的生命交在我的医生手里 |  |  |  |  |  |
| All in all, I have complete trust in my doctor.  总之，我完全信任我的医生 |  |  |  |  |  |
| If I give the physician extra money or gifts, I will receive better care.  如果我给我的医生钱或礼物，我会得到较好的照顾 |  |  |  |  |  |
| The cost of medical care is a burden on me.  医疗费用对我是一个重担 |  |  |  |  |  |

1. What reasons, if any, affect your trust towards your physician? 是否可以解释不信任医生的原因？____________________________________________________________________________________________________________________________________________________
